# Staged uniportal video-assisted thoracoscopic bilateral lower lobectomy for bilateral intralobar pulmonary sequestration complicated by *Aspergillus* infection: a case report

**DOI:** 10.3389/fsurg.2026.1875717

**Published:** 2026-06-11

**Authors:** Dongjie Ma, Yang Song, Yicheng Liang, Xiaohua Shi, Hongsheng Liu, Zhiyong Zhang, Shanqing Li

**Affiliations:** 1 Department of Thoracic Surgery, Peking Union Medical College Hospital, Chinese Academy of Medical Sciences & Peking Union Medical College, Beijing, China; 2 Department of Pathology, Peking Union Medical College Hospital, Chinese Academy of Medical Sciences & Peking Union Medical College, Beijing, China

**Keywords:** *aspergillus* infection, bilateral pulmonary sequestration, case report, isthmic bridge, three-dimensional reconstruction, uniportal VATS

## Abstract

**Background:**

Bilateral pulmonary sequestration (BPS) is an exceptionally rare congenital bronchopulmonary and vascular anomaly. Its complex anatomy and aberrant systemic arterial supply make diagnosis and treatment difficult, especially when severe fungal infection is present.

**Case presentation:**

A 28-year-old woman presented with a 1-year history of cough and hemoptysis and bilateral lower-lung masses. CT with 3D reconstruction showed bilateral intralobar pulmonary sequestration linked by an anomalous fistulous tract across the posterior mediastinum and supplied by multiple arteries from the descending aorta. Sputum culture and bronchoalveolar lavage next-generation sequencing confirmed *Aspergillus* infection. Because systemic antifungal therapy was ineffective, surgery was performed. Guided by 3D reconstruction, staged uniportal video-assisted thoracoscopic bilateral lower lobectomy was chosen to maximize safety and preserve function. The more severely infected left lower lobe was resected first, followed by pulmonary rehabilitation. Six months later, the right lower lobectomy and careful division of the communicating tract were completed successfully. Histopathology confirmed bilateral intralobar pulmonary sequestration and *Aspergillus* hyphae in the bronchial lumen. One year after surgery, lung expansion and cardiopulmonary function were well preserved, with no major postoperative complications and an excellent overall recovery.

**Conclusion:**

This case delineates the clinical presentation of an extremely rare bilateral ILS connected via a posterior mediastinal fistulous tract and complicated by aspergilloma. Staged uniportal VATS, augmented by preoperative 3D reconstruction and interval pulmonary rehabilitation, represents an optimal, safe, and effective strategy for managing such intricate congenital malformations while maximizing functional preservation.

## Introduction

Pulmonary sequestration (PS), first systematically defined by Pryce in 1946, is a rare congenital anomaly characterized by non-functioning dysplastic lung tissue that lacks normal communication with the tracheobronchial tree and receives anomalous systemic arterial blood supply (1). It accounts for merely 0.15% to 6.4% of all congenital pulmonary malformations (2, 3). Morphologically, PS is classified into intralobar sequestration (ILS) and extralobar sequestration (ELS). ILS, constituting 75% to 85% of cases, shares the visceral pleura of the adjacent normal lung and typically drains into the pulmonary venous system. ILS occurs almost exclusively in the lower lobes (98%), with about 55% localizing to the left lung (1, 4). According to Pryce's classic classification, intralobar sequestration is divided into three types based on the relationship between the anomalous systemic artery and the pulmonary tissue: Type I, in which an anomalous systemic artery supplies normal lung tissue without bronchial sequestration; Type II, in which the anomalous artery supplies both normal and sequestered bronchopulmonary tissues; and Type III, in which the anomalous artery exclusively supplies the sequestered, non-functioning lung tissue that lacks normal communication with the tracheobronchial tree (1). Although pathologically benign, PS frequently leads to severe clinical complications, including recurrent pulmonary infections, hemoptysis, ventilation-perfusion mismatches, and rarely, congestive heart failure resulting from left-to-left shunting (5).

Bilateral pulmonary sequestration (BPS) is phenomenally rare, with only a few sporadic cases documented in the literature (6–8). The concurrent presence of an anomalous communicating fistulous tract bridging the bilateral sequestered lobes across the posterior mediastinum, further complicated by an opportunistic fungal infection (e.g., *Aspergillosis*), makes accurate anatomical mapping and surgical planning profoundly challenging. Guided by the CARE (CAse REports) clinical reporting guidelines (9), we herein present an extraordinary case of adult bilateral ILS connected by a mediastinal sinus tract and complicated by refractory *Aspergillus* infection. We elaborate on the rationale, strategic planning, and successful execution of a staged uniportal VATS bilateral lower lobectomy assisted by preoperative 3D reconstruction.

## Case presentation

### Patient information and clinical findings

A 28-year-old female patient was admitted to our institution with a 1-year history of spontaneous cough and blood-streaked sputum. She denied any history of smoking, pet ownership, exposure to specific occupational physicochemical hazards, or a relevant family history of congenital malformations. Physical examination revealed a normal body temperature, stable vital signs, and a peripheral capillary oxygen saturation (SpO2) of 99% on room air. Respiratory movements were symmetrical. Palpation and percussion were unremarkable, and auscultation revealed no obvious dry or wet rales. No vascular murmurs were audible over the chest wall.

### Diagnostic assessment

**Figure 1:**
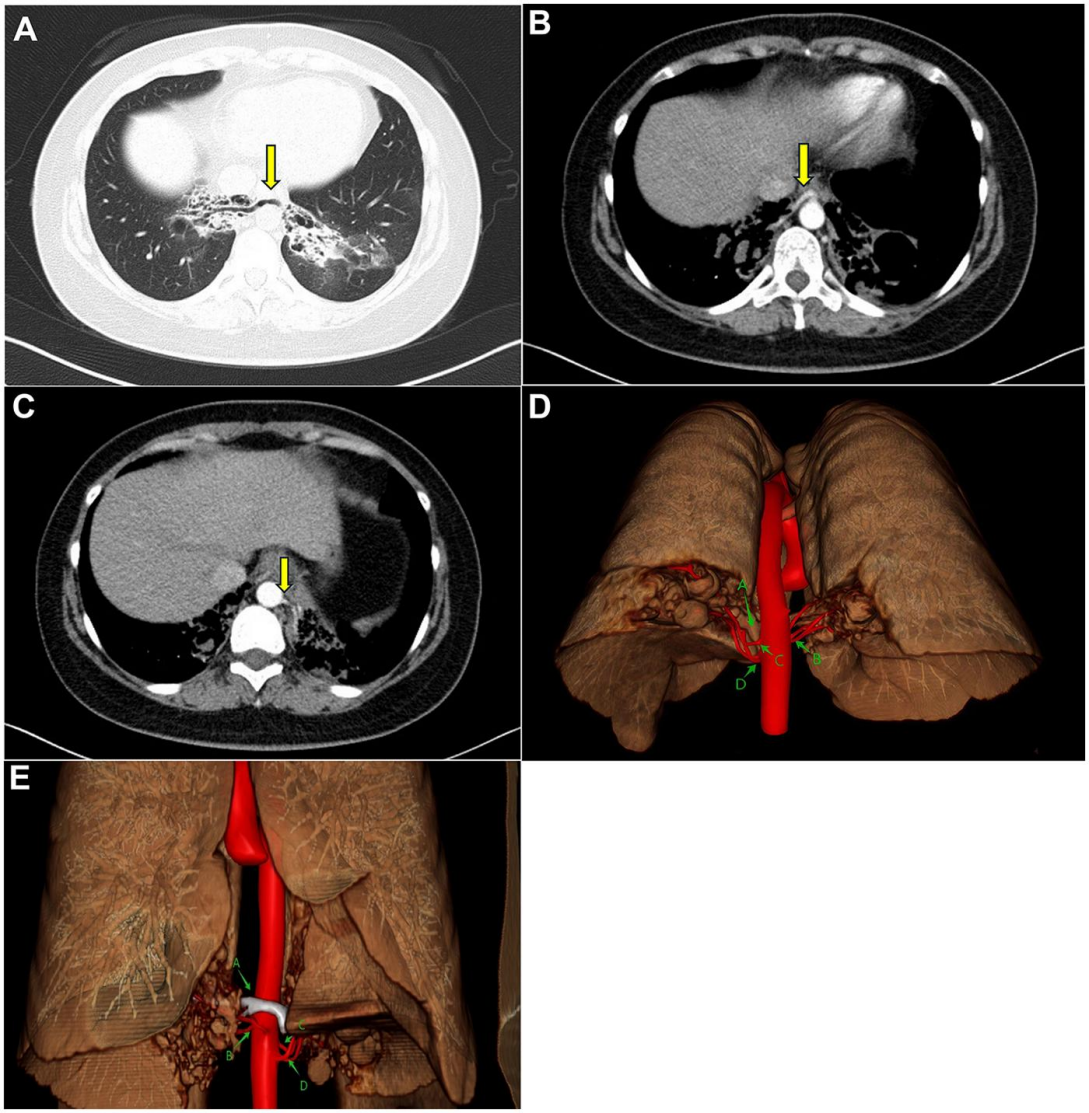
Preoperative computed tomography (CT) imaging and three-dimensional (3D) reconstructions. **(A)** Enhanced CT lung and mediastinal windows demonstrating the continuous tubular structure connecting the bilateral lower lobe lesions. **(B)** CT scan identifying the aberrant systemic artery supplying the right lower lobe. **(C)** CT scan identifying the aberrant systemic artery supplying the left lower lobe. **(D)** 3D reconstruction clearly depicting the pipeline structure (labeled A) connecting the bilateral lesions, alongside the aberrant systemic arteries (labeled B, C, D) arising from the descending aorta. **(E)** Posterior view of the 3D reconstruction detailing the anatomical relationship between the isthmic bridge and the anomalous arteries.

In September 2024, a contrast-enhanced chest CT scan revealed bilateral paraspinal patchy opacities in the lower lobes, containing multiple air-filled cystic cavities and soft tissue nodules with a characteristic “air crescent sign.” Crucially, the lung and mediastinal windows of the CT identified a continuous tubular fistulous tract connecting the left and right cystic lesions across the posterior mediastinum (Figure 1A). Furthermore, the CT directly visualized a robust aberrant systemic artery supplying the right lower lobe (Figure 1B) and another anomalous artery supplying the left lower lobe (Figure 1C).

Subsequent 3D reconstructions precisely mapped the spatial anatomy. The reconstructed models clearly demonstrated the anomalous pipeline structure (labeled A) connecting the bilateral lesions, alongside the complex origins and trajectories of the multiple aberrant systemic arteries (labeled B, C, D) arising from the descending thoracic aorta (Figures 1D,E). Additional 3D angles further corroborated the frontal and detailed views of the communicating fistulous tract alongside the solid and cystic nodules (Supplementary Figure S1A). Correlating these findings with Pryce’s classification, the patient’s anatomical presentation was classified as bilateral Pryce Type III intralobar pulmonary sequestration (ILS), as multiple robust anomalous systemic arteries arising from the descending thoracic aorta exclusively supplied the non-functioning, cystic-subverted bilateral lower lung lobes. Whole-body PET/CT imaging indicated mildly increased radiotracer uptake within these lesions (SUVmax 2.7). This examination was specifically conducted to rule out potential underlying primary or secondary pulmonary malignancy, given the highly atypical bilateral mass-like presentation with solid soft-tissue components, and to assess the metabolic activity and extent of the severe active infectious process. Sputum culture and next-generation sequencing (NGS) of the bronchoalveolar lavage fluid confirmed an active *Aspergillus* infection. Following multidisciplinary consultations involving the Departments of Infectious Diseases and Thoracic Surgery, conservative antifungal therapy was deemed ineffective, and surgical resection of the sequestered lungs was recommended.

### Therapeutic intervention (staged surgery)

Given the extensive involvement of the BPS and the severe infection, a simultaneous bilateral lobectomy carried prohibitive risks of acute respiratory distress. Thus, a staged uniportal VATS strategy was formulated utilizing 3D preoperative planning. Preoperative endovascular embolization of the aberrant systemic arteries was thoroughly considered but was ultimately not performed for two primary reasons. First, our preoperative 3D reconstruction provided an exceptionally clear vascular roadmap, allowing safe, direct VATS dissection and anatomical stapling of the anomalous high-pressure vessels under direct vision without requiring endovascular assistance. Second, endovascular embolization can induce significant perivascular aseptic inflammation, local edema, and subsequent fibrosis, which often obliterates the surgical cleavage planes around the descending thoracic aorta, potentially increasing the technical difficulty and hemorrhagic risk of subsequent VATS dissection.

After ruling out surgical contraindications, the first-stage surgery was performed on September 21st, 2024. The patient underwent a uniportal VATS left lower lobectomy, prioritizing the resection of the more severely infected left lower lobe. During the first-stage left lower lobectomy, intraoperative exploration revealed a thick, fibrous tubular tract (isthmic bridge) measuring approximately 1.5 cm in diameter, crossing the posterior mediastinum from the left-sided sequestered lung tissue to the contralateral side. The tract was carefully mobilized from the surrounding loose connective tissues of the posterior mediastinum, clamped close to the mediastinal pleura, and transected using an endoscopic linear stapler, leaving a securely closed blind-ending stump on the right side. Intraoperative exploration, vessel ligation, and anomaly transection proceeded smoothly. The patient recovered well, and over the subsequent six months, she underwent a prolonged course of targeted oral antifungal therapy (Voriconazole, 200 mg orally twice daily) for a total of 3 months to eradicate any residual fungal burden and prevent postoperative pleural or systemic dissemination. Simultaneously, she underwent intensive physical and cardiopulmonary prehabilitation to optimize cardiopulmonary reserves. This extended 6-month interval was deliberately chosen over the standard 1–2-month inter-stage period to facilitate comprehensive systemic recovery, allow complete functional compensation of the remaining lung tissue, and ensure stable, infection-free healing of the transected mediastinal communicating tract stump before the contralateral major lobectomy.

**Figure 2:**
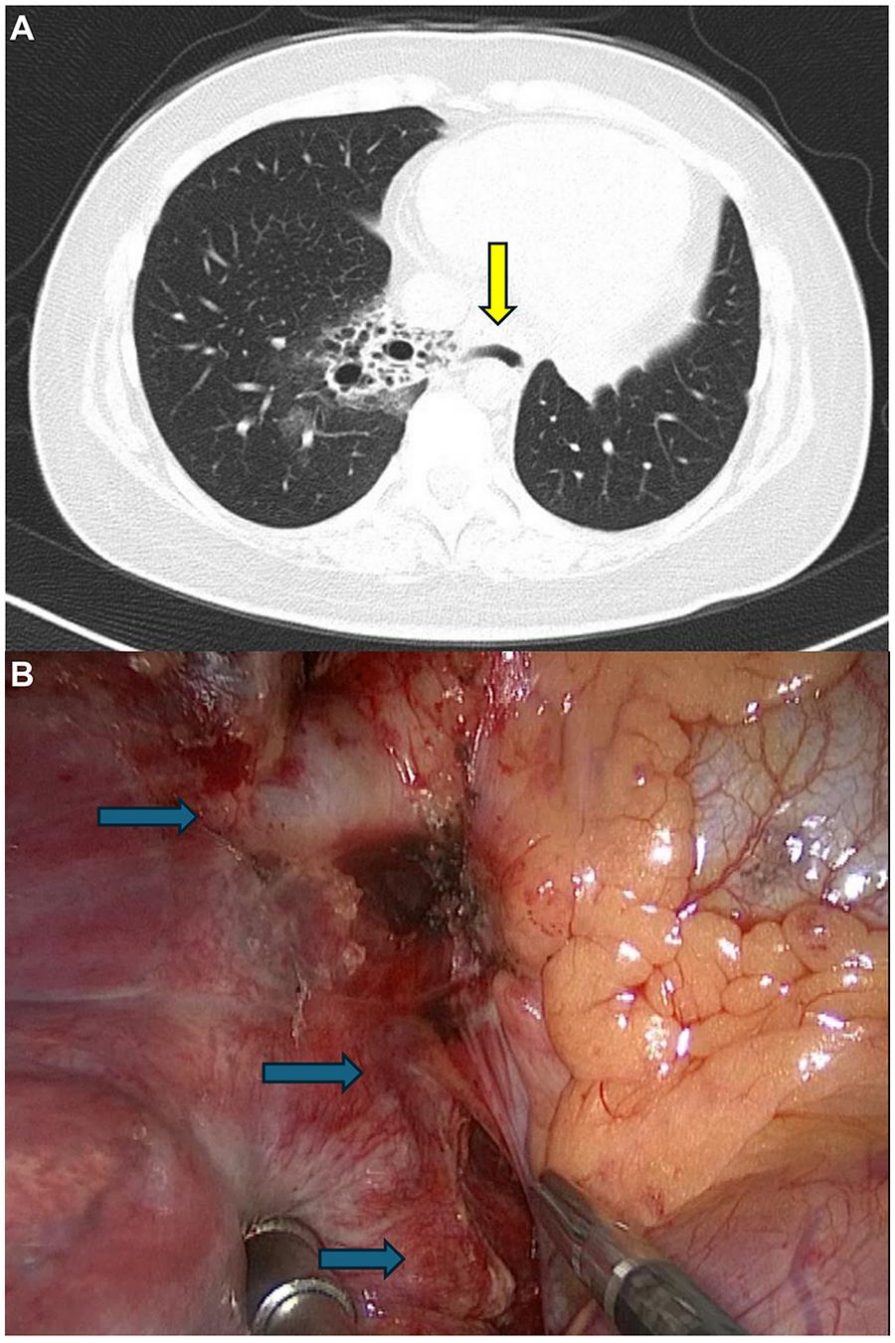
Inter-stage main radiologic evaluation and intraoperative surgical view. **(A)** Follow-up contrast-enhanced CT before the second-stage surgery (March 2025) showing the blind end of the left fistulous tract (indicated by arrow). **(B)** Intraoperative view during the right lower lobectomy (cranial to caudal perspective). The arrows sequentially demonstrate the right inferior pulmonary vein, the communicating sinus tract, and the right anomalous systemic artery.

In March 2025, an interval enhanced CT scan confirmed that the residual fistulous tract in the posterior mediastinum ended blindly with small air spaces and no active infection (Figure 2A). Additional inter-stage detailed scans confirmed the status of the right cystic lesions and the patency of the remaining right aberrant artery (Supplementary Figures S1B–D). Preoperative pulmonary function tests showed well-compensated capacities (Table 1). Consequently, the second-stage surgery (uniportal VATS right lower lobectomy) was conducted on March 12th, 2025. Utilizing the precise 3D roadmap, anatomical structures were dissected and stapled in a unidirectional, cranial-to-caudal sequence. Intraoperative exploration clearly exposed the sequential layout from cranial to caudal: the right inferior pulmonary vein, the communicating sinus tract, and the aberrant systemic artery derived from the aorta (Figure 2B). The residual portion of the communicating sinus tract was identified as a fibrous, blind-ending cord extending from the posterior mediastinum into the right-sided sequestration. Surgical dissection confirmed it did not communicate with the esophagus, tracheobronchial tree, or pericardium. The tract was dissected down to its mediastinal origin, securely ligated at its base, and completely excised in block with the right lower lobe specimen to prevent any risk of fluid accumulation or mediastinal infection. The patient was discharged smoothly on the second postoperative day without complications.

**Table 1:** Pulmonary and cardiac function parameters across the treatment timeline.

Parameter	Stage 1 preop (Sept 2024)	Stage 2 preop (Mar 2025)	1-Year follow-up (Mar–Apr 2026)
FEV1 (L) [% predicted]	2.90 (86.0%)	2.75 (81.0%)	2.57 (77.7%)
FVC (L) [% predicted]	3.35 (87.0%)	3.17 (82.0%)	2.91 (76.9%)
FEV1/FVC (%)	86.56%	86.66%	83.03%
DLCO/VA (% predicted)	1.56 (85.0%)	1.68 (93.0%)	1.60 (88.5%)
LVEF (%) [Echocardiography]	Not Assessed	Not Assessed	61.0% (Normal structure)

### Pathological findings

**Figure 3:**
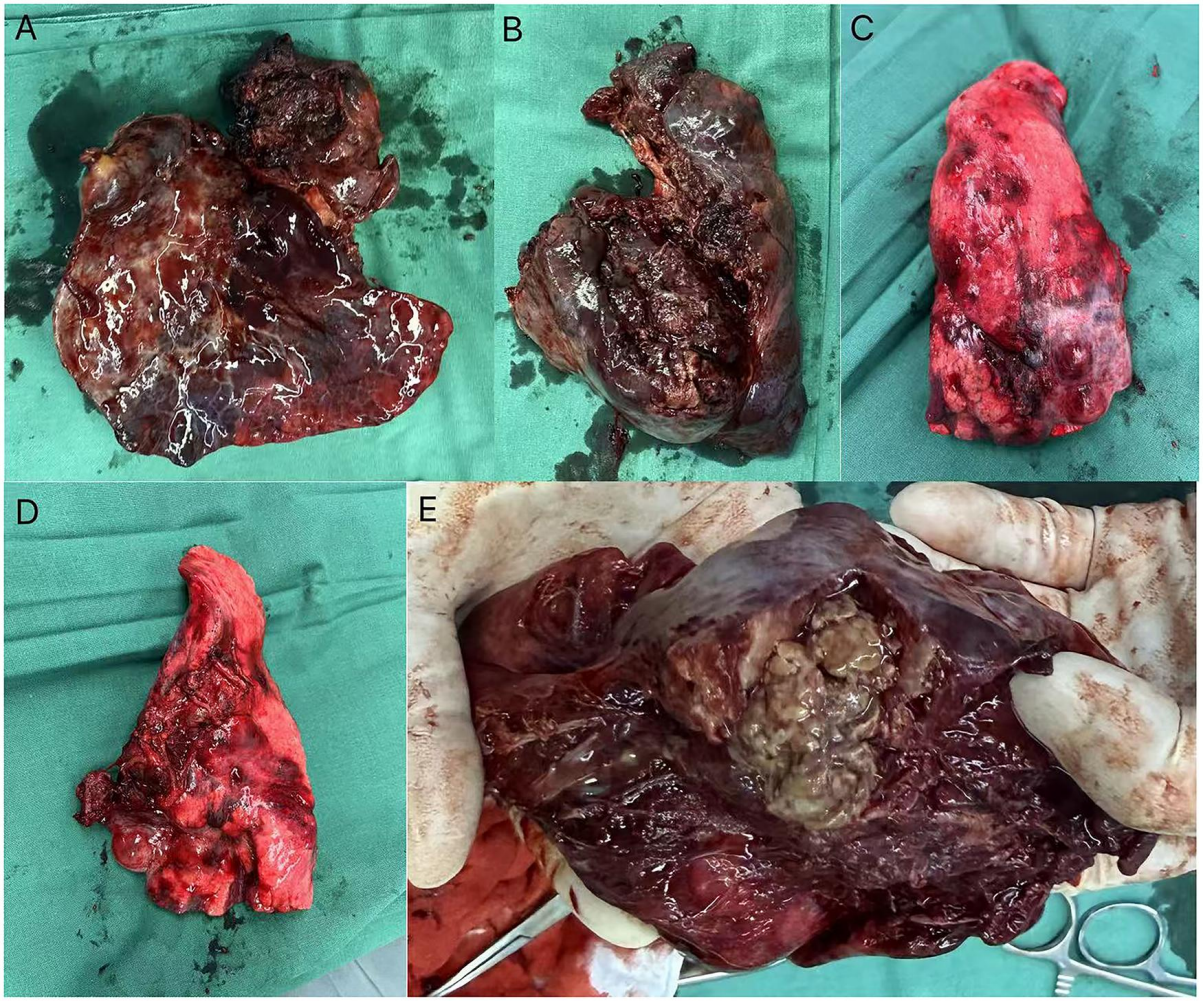
Macroscopic pathology of the resected bilateral pulmonary sequestered lobes. **(A)** Ventral view and **(B)** dorsal view of the resected right lower lobe. **(C)** Ventral view and **(D)** dorsal view of the resected left lower lobe. **(E)** Cross-section of the right lower lung cavity revealing a distinct *Aspergillus* fungal ball nestled within the disrupted parenchyma.

Macroscopic examination of the resected bilateral lower lobes displayed consolidated lung parenchyma with multilocular cysts. The ventral and dorsal perspectives of the right lower lobe (Figures 3A,B) and left lower lobe (Figures 3C,D) were comprehensively documented. Notably, a distinct *Aspergillus* fungal ball was visually identified within the right lower lobe cavity upon cross-section (Figure 3E).

**Figure 4:**
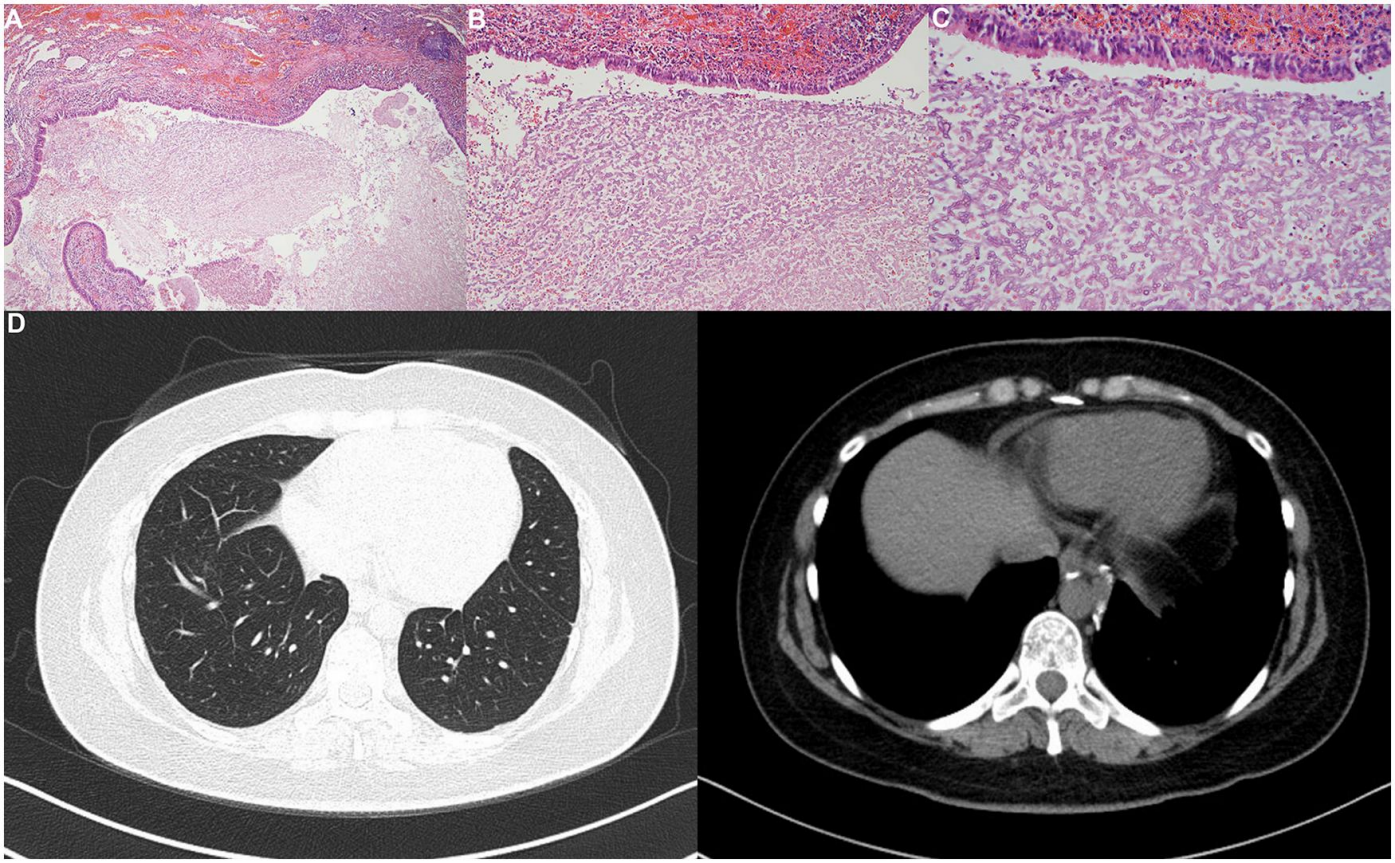
Microscopic histopathology and long-term follow-up imaging. **(A)** Low-magnification histopathology (H&E, 4 ×) illustrating chronic structural distortion and cystic bronchial dilation. **(B)** Medium-magnification view (H&E, 10 ×) showing inflammatory infiltration. **(C)** High-magnification microscopy (H&E, 20 ×) providing clear, definitive evidence of abundant, acutely branching *Aspergillus* hyphae. **(D)** Follow-up CT scan (September 2025) demonstrating complete bilateral lung expansion without pleural effusion.

Formalin-fixed paraffin-embedded histopathological analysis (H&E staining) confirmed the diagnosis of bilateral intralobar pulmonary sequestration characterized by chronic inflammation and cystic architectural distortion. Microscopic examination at a low magnification (4 ×) illustrated the extensive destruction of the bronchial walls and cystic dilations (Figure 4A). Progressive medium (10 ×) and high-resolution (20 ×) microscopic examinations unequivocally demonstrated an abundance of acutely branching fungal hyphae within the dilated bronchial lumina, providing definitive evidence of the aspergilloma (Figures 4B,C). Sampled peribronchial lymph nodes exhibited reactive chronic inflammation (right: 0/3; left: 0/9), with no signs of malignancy.

### Follow-up and outcomes

A follow-up CT scan on September 25th, 2025, demonstrated satisfactory bilateral pulmonary expansion, absence of pleural effusion, and a benign high-density postoperative change in the posterior mediastinum (Figure 4D). Comprehensive functional evaluations conducted in March and April 2026 (one year after the completion of bilateral lobectomies) yielded excellent results. Pulmonary function tests recorded an FEV1 of 2.57 L (77.7% predicted) and a robust diffusing capacity (DLCO/VA) of 88.5% predicted. Furthermore, resting transthoracic echocardiography confirmed normal intracardiac structures with a preserved left ventricular ejection fraction (LVEF) of 61% (Table 1), underscoring the success of the staged surgical strategy.

## Discussion

The etiology of pulmonary sequestration primarily traces back to the aberrant budding of the embryonic foregut (4). However, the specific clinical morphology of the patient in this report incorporates three extraordinary rarity elements that substantially amplified the clinical difficulty: (1) symmetrical bilateral presentation of intralobar sequestration; (2) the existence of a direct communicating fistulous tract (isthmic bridge) traversing the posterior mediastinum; and (3) a profound, anatomically entrenched *Aspergillus* fungal ball infection culminating in recurrent hemoptysis.

BPS comprises an infinitesimal fraction of all PS diagnoses. Wei et al. reviewed 2,625 PS cases and identified only 3 patients with bilateral involvement (10). BPS can present simultaneously as intralobar and extralobar types. Among 10 documented cases with concurrent ILS and ELS, 8 had the ILS on the right and 2 on the left, whereas all ELS lesions were situated on the left (11). While an “isthmic bridge” (or saddlebag configuration) connecting bilateral sequestrations has been sporadically reported in the pediatric demographic (12, 13), it is virtually unprecedented in an adult complicated by fungal spherules. The sequestered lung tissues, devoid of native tracheobronchial clearance, serve as an ideal nidus for opportunistic pathogens. Once an aspergilloma colonizes these cystic spaces, conventional antifungal pharmacotherapy is rendered almost entirely impotent due to the avascular nature of the cystic cavity interior, strictly dictating surgical extirpation as the sole definitive remedy (14).

Although sublobar resections (e.g., segmentectomy) or endovascular embolization are frequently favored for localized, uninfected PS to spare functional parenchyma or minimize bleeding risks (15), such conservative approaches were contraindicated in this patient. The diffuse architectural destruction wrought by the multilocular cysts, combined with the entrenched fungal burden, necessitated complete anatomical lobectomies to avert catastrophic postoperative complications like fungal empyema or intractable bronchopleural fistulas.

Indeed, surgical resection for chronic, complex pulmonary infectious processes—particularly those complicated by invasive or localized *Aspergillus* colonization—is fraught with technical challenges and high complication rates. Long-standing fungal infection often induces dense, highly vascular pleural adhesions, severe hilar lymphadenopathy, and chronic parenchymal architectural distortion that can completely subvert normal anatomical landmarks. In extreme scenarios, such complex infections can necessitate extended resections, such as extrapleural pneumonectomy, carinal resection, or tracheal sleeve pneumonectomy to completely eradicate the diseased tissues (16). Therefore, meticulous preoperative design and flawless surgical execution are paramount to minimize infectious and fistulous surgical complications, thereby avoiding any forced, extended parenchymal resections beyond the bilateral lower lobes. In our patient, the potential for catastrophic postoperative complications, such as fungal empyema, bronchopleural fistula, or massive hemorrhage from the high-pressure anomalous systemic arteries, was significantly mitigated by utilizing the staged, uniportal VATS strategy augmented by preoperative 3D mapping.

Crucially, executing simultaneous bilateral lobectomies risks precipitating severe right ventricular strain and respiratory failure. Therefore, we deliberately orchestrated a staged uniportal VATS lobectomy framework. Preoperative 3D CT reconstruction was indispensable in navigating this complex anomaly. By rendering a spatial map of the highly variable aberrant systemic arteries and the exact trajectory of the communicating sinus tract, it empowered the surgical team to formulate a precise approach angle, secure these anomalous high-pressure vessels safely, and transect the communicating tract without blind exploration (17). The strategic six-month hiatus, reinforced by dedicated pulmonary rehabilitation, successfully buffered the physiological impact, culminating in stellar long-term functional adaptation as evidenced by a sustained DLCO/VA of 88.5% and normalized cardiac dynamics.

## Conclusion

This report elucidates the successful management of a profoundly rare case of adult bilateral intralobar pulmonary sequestration interconnected by a posterior mediastinal sinus tract and severely complicated by an *Aspergillus* infection. It strongly advocates that a staged uniportal VATS lobectomy approach, precisely guided by 3D anatomical reconstruction and complemented by structured inter-stage physical rehabilitation, constitutes a safe, minimally invasive, and functionally optimal therapeutic paradigm for highly complex congenital lung malformations.

## Data Availability

The original contributions presented in the study are included in the article/Supplementary Material, further inquiries can be directed to the corresponding author.

## Supplementary material

Supplementary Figure S1 Additional 3D and Inter-stage CT Imaging. **(A)** Frontal view of the 3D reconstruction demonstrating the paraspinal cavitary lesions and the communicating fistulous tract. **(B)** Inter-stage CT detailing the right lower lobe cystic/solid lesions. **(C)** Inter-stage CT magnifying the location of the connecting sinus tract structure. **(D)** Inter-stage CT confirming the right-sided aberrant systemic artery prior to the second-stage surgery.
