# Supplementary figures and images for: Staged uniportal video-assisted thoracoscopic bilateral lower lobectomy for bilateral intralobar pulmonary sequestration complicated by Aspergillus infection: a case report
Source: Front Surg. 2026 Jun 11;13:1875717. doi: 10.3389/fsurg.2026.1875717 (PMC13294214; doi:10.3389/fsurg.2026.1875717)

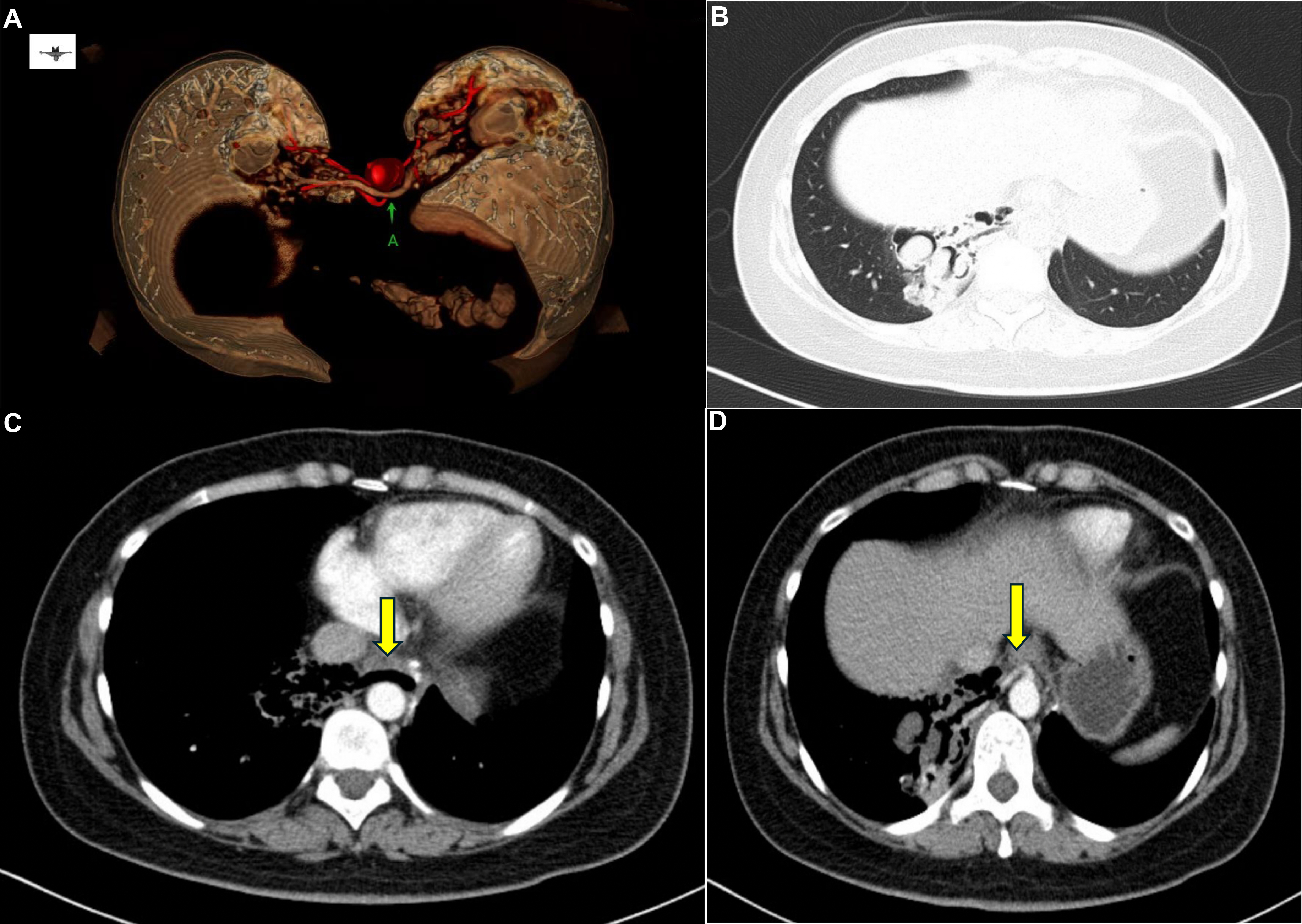

Supplement: Supplementary Figure S1 — Additional 3D and Inter-stage CT Imaging. (A) Frontal view of the 3D reconstruction demonstrating the paraspinal cavitary lesions and the communicating fistulous tract. (B) Inter-stage CT detailing the right lower lobe cystic/solid lesions. (C) Inter-stage CT magnifying the location of the connecting sinus tract structure. (D) Inter-stage CT confirming the right-sided aberrant systemic artery prior to the second-stage surgery. [file Image1.jpeg]
